# Four-Screw Plate Fixation vs Conventional Fixation for Diaphyseal Fractures of the Forearm

**DOI:** 10.5812/traumamon.4497

**Published:** 2012-05-26

**Authors:** Seyed Abdolhossein Mehdi Nasab, Nasser Sarrafan, Saeed Sabahi

**Affiliations:** 1 Department of Orthopaedic Surgery, Imam Khomeini Hospital, Jundishapur University of Medical Sciences, Ahvaz, IR Iran

**Keywords:** Forearm, Fracture, Fixation

## Abstract

**Background:**

Standard treatment of diaphyseal fractures of the forearm is open reduction and fixation using dynamic compression plates (DCP) and screws. This technique uses screw placement in all 6 or more of the plate holes except the hole over the fracture line. We hypothesized that DCP with selective 4-screw bicortical placement can provide adequate fixation for these fractures.

**Objectives:**

The aim of this study was to evaluate the results of conventional 6 or more screw fixation versus 4 screw fixation for adults with diaphyseal fractures of the forearm.

**Patients and Methods:**

In this prospective study, 128 fractures of the ulna, radius or both bones of the forearm in 87 patients were treated in either one of these two groups: Open reduction and internal fixation (ORIF) with conventional DCP and screws or ORIF using DCP and selective 4- screw placement. Fractures were transverse or oblique in pattern without gross comminution. In a total of 41 patients with fractures, 28 single ulnar and 18 single radius fractures were included. Follow-up visits were done at 3-6 and 12-16 weeks and at 6 months. Outcome with respect to union an nonunion rates, union time, infection, and device failure was noted.

**Results:**

No change in alignment was noted in any patient. Union time in conventional and selective bicortical 4-screw fixation was 74.8 days and 73.6 days respectively which showed no significant difference (P = 0.064). Union rate and infection was 92.1% and 3.2% in conventional and 95.3% and 0% in the selective group respectively. Non-union was observed in 5 and 3 cases of fractures in conventional and the selective group respectively.

**Conclusions:**

For treatment of the transverse or oblique diaphyseal fractures of the forearm, fixation by a same length 3.5 mm DCP with selective 4-screw cortical fixation (2 screws on each side of the fracture site) had similar results in comparison with conventional 6 or more DCP screws. Because of lesser impact on host bone and smaller incision, the selective 4-screw insertion can be an alternative technique for treatment of these fractures.

## 1. Background

The forearm has an important role in the function of the upper extremity thus, loss of forearm motion resulting from a poorly treated fracture can be disabling. The preferred method for diaphyseal fracture of the forearm is ORIF using plate and screws. Selection of the plate type is controversial as some investigators prefer LCP, whereas most of the others recommended DCP and have reserved LCP for those who have fractures in the metaphysis or have osteoporotic bone. Anatomic reduction allows maintaining normal alignment of the length and distal or proximal radioulnar joints and restoration of normal supination and pronation functions(1-4).

Anderson et al has reported that plating is the most physiologic type of fixation for forearm shaft fractures. In his study with 330 acute forearm diaphyseal fractures, 96.3% union in ulnar and 97.8% in radial fractures with compression plates were reported (1). Chapman et al. found 90% union in 129 diaphyseal fractures treated by compression plating (3).

Although open reduction and internal fixation with DCP is the most commonly accepted treatment method for adult forearm diaphyseal fractures, however, this technique has some disadvantages such as extensive soft tissue or periosteal damage and refracture after plate removal (2, 5, 6). The rate of this complication after plate removal has been reported between 4% to 25% (7, 8). This complication is related to the size of plate, surgical technique, stress from screw holes, bone atrophy or osteonecrosis of the cortex under the plate (1, 3, 9). In conventional plating, screw placement is used in all holes of plate, except at the fracture line. Though the new plates such as LC- DCP are used to minimize periosteal stripping, there is a tendency to use the least hardware. This may facilitate a more physiologic process for fracture healing by less damage to the local soft tissue and bone (10-12). Mast et al. suggested that selective insertion of screws in the bone is possible and stated: “Every screw must have its own special function” (13). According to this concept, we felt that fewer screws at each site of fracture with standard plate length may decrease the complications related to more screw placement in conventional DCP. This can be true in nonweight-bearing bones such as radius or ulna. There are few reports for selective 4 screw insertion in forearm fractures and a rate of union in more than 90% of the cases has been reported (14, 15). The choice of plate will depend on the size of the bone and pattern of fracture. A 3.5 mm DC plate or a narrow 4.5 mm plate is widely used to fix forearm fractures. The length of plate is ultimately dependent on the degree of fracture comminution, and fixation of at least 6 cortices is used in conventional forearm plating (16). Emphasis on this point of view is important that although the insertion of all screws in a standard plate can have more stability, however, more periosteum or cortical damage can occur during drilling of the bone.

## 2. Objectives

The purpose of the present study was to evaluate the hypothesis that use of selected screw insertion in a standard length DC plate for treatment of forearm diaphyseal fractures can provide enough stability, enhance bone healing and decrease complication rates when compared with use of conventional 6 or more hole fixation plates.

## 3. Patients and Methods

This prospective cross-sectional study was performed between Sept 2007 and Aug 2010 at two university hospitals (Imam Khomeini & Razi) in Ahvaz Iran. From Sept 2007 to Oct 2009, our protocol was open reduction and internal fixation for forearm fractures using a 3.5 mm DCP with 6 cortical screws. We treated forearm fractures with the same type of plate but inserted 4-selective screw holes. If we felt that by this technique rigid fixation could not be achieved, then we operated as per the routine method and the patient was excluded from study. The study was approved by the ethics committee at our university and informed consent was taken from all patients.

Adult patients over 18 years-old with closed fracture of the radius, ulna or both bones of the forearm that were operated by senior residents were included. The fractures were classified according to OTA classification system. Type A or B fractures were treated by open reduction and internal fixation (ORIF) using standard length of plate. Type C or open fractures were excluded. Patients were operated in a mean time of 6 ± 3 days after fractures and selected for either of the 2 groups: 1- ORIF with conventional plate and 3 or more screw fixation at each side of the fracture site. 2- ORIF with selective 2 screw fixation at each side of the fracture. A single dose 1 gr cephalothin was given before operation and continued for 24-48 hours.

Standard approaches of volar or dorsal for radius and direct subcutaneous approach for the ulna was used. A 3.5 mm DCP and screw was used for fixation in all fractures. The same protocol including posterior splinting for 3 weeks for pain relief and stitch removal and 20 sessions of physiotherapy was advocated for all subjects. They were allowed finger mobilization on the 1st day after operation. Gentle active motion of the wrist and elbow was encouraged after splint removal; however, vigorous heavy activities were delayed until union was observed upon radiography. Follow-up visits were performed and radiographs were taken at 3, 6, 12, 16 and 20 weeks and 6 months after operation. Union was defined as complete obliteration of fracture gap on 2 perpendicular view radiographs. Delayed or nonunion was considered when fracture gap was present or absence of progressive callus formation by 6 months was seen. The patients were assessed for rate and time to fracture healing, device failure and early complications. Statistical analysis was performed using t-test and Chi-square tests with SPSS version 13 software; significant difference was considered when the P value was less than 0.05.

## 4. Results

Between 2006 and 2010, 87 patients with 128 fractures of the ulna, radius or both bones were included in the study. Mean age was 34.9 ± 12.5 years. There were 28 (32.1%) female and 59 (67.9%) male patients. The cause of fractures were traffic accidents in 48 patients, direct trauma in 18 and falls in 21 patients. The most frequent DCP had 7-holes used in 86 fractures (67.2%). Table 1 shows the plates used for fractures. The overall union rate in all fractures was 74.05 ± 9.6 days.

**Table 1.** Types of plates used in both groups

Method of treatment	Plate 6 holes	Plate 7 holes	Plate 8 holes	Plate 9 holes	Total
Conventional plating, No, %	26, 37.6	38, 55.2	5.8 ,4	1, 1.5	69, 100
Selective screw fixation, No, %	7, 11.7	48, 81.5	2, 3.4	2, 3.4	59, 100
Total, No, %	33, 25.7	86, 67.2	6, 4.7	3, 2.4	128, 100

The mean operating time was 63 minutes in selected screw group and 76 minutes in the conventional group with no significant difference P = 0.06. The average union time in all patients was 74.2 ± 9.6 days (54-98 days). In the selected screw group, the mean union time was 73.6 ± 8.8 days whereas in conventional screw group was 74.8 ± 10.4 days which showed no significant difference (P = 0.064) ; 95.3% of the 4-screw fractures healed by 6 months; this was 92.1% for the 6- screw group. In terms of union rate, no significant difference was noted between the groups (P = 0.811, Table 2, 3). Nonunion was observed in 3 (4.6%) fractures in the 4-screw group and in 5 cases (7.8%) of the conventional group. No significant difference was seen in terms of nonunion (P = 0.053).

**Table 2.** Results in both groups of patients

Method of treatment	Union time, Day	Rate of union, n , %	Infection, n, %
Conventional plating	74.8 ± 10.4	64, 92.76	2, 2.90
Selective screw fixation	73.6 ± 8.8	56, 95	0
P value	0.064	0.811	0.048

**Table 3.** Results of treatment in men and women

Sex of Pts ^a^	No of Pts ^a^	No of Fx ^a^	No Con ^a^ screw	No Sel ^a^ Screw	Time of union, days	Rate of union, %	Non union, %	Infection, n, %
Male	59	86	47	39	74.8 ± 10.2	96.4	2.1	1, 1.45
Female	28	42	24	18	73.6 ± 8.5	95.2	3.1	1, 1.45
Total	87	128	71	57	73.2 ± 28	95.6	5.2	2, 2.9

^a^ Abbreviations: Con: conventional; Fx: fractures; Pts: patients; Sel: selective;

**Figure 1.**
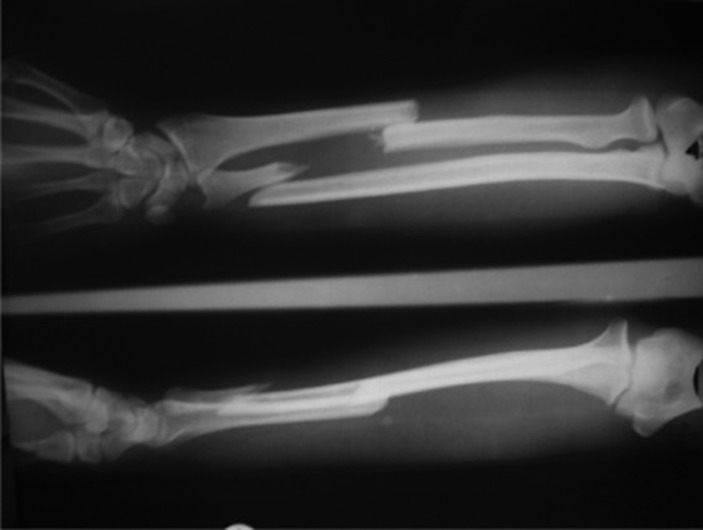
Fracture of the radius and ulna in a 42 year-old man.

**Figure 2.**
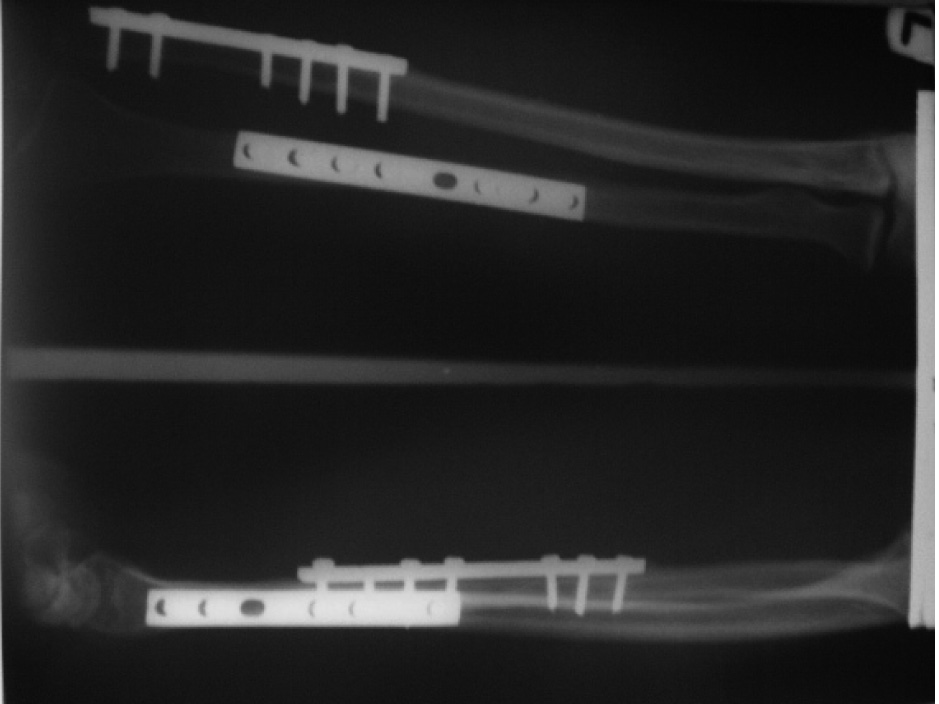
Fixation of both bones with conventional DCP.

**Figure 3.**
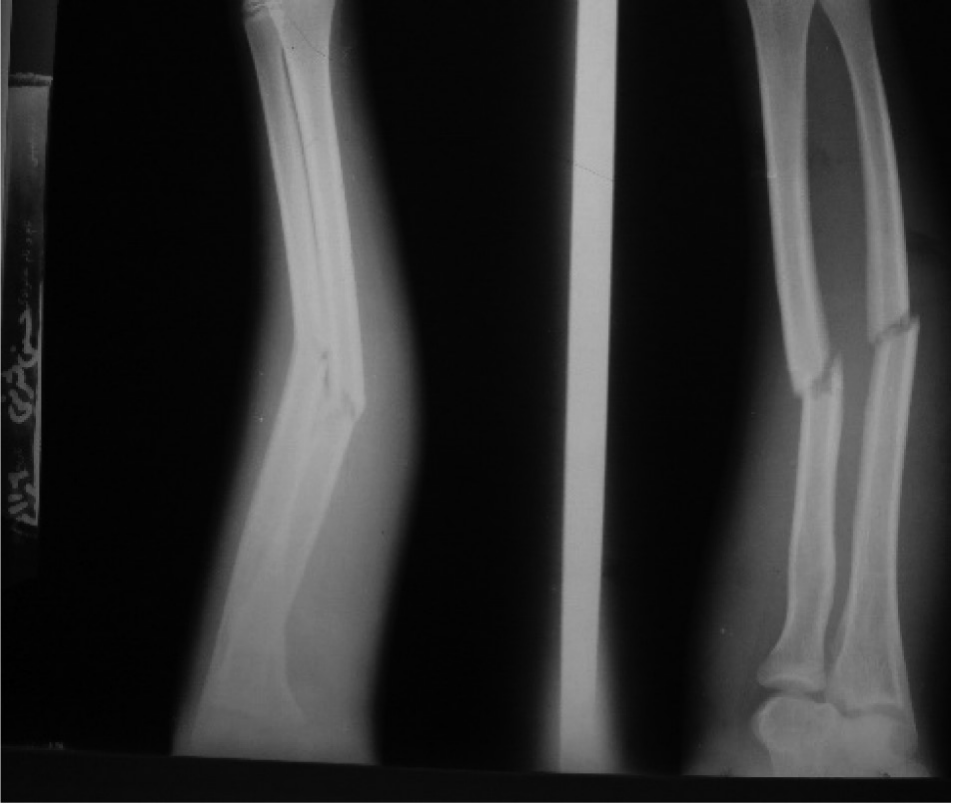
Fracture of the ulnar and radius diaphysis.

**Figure 4.**
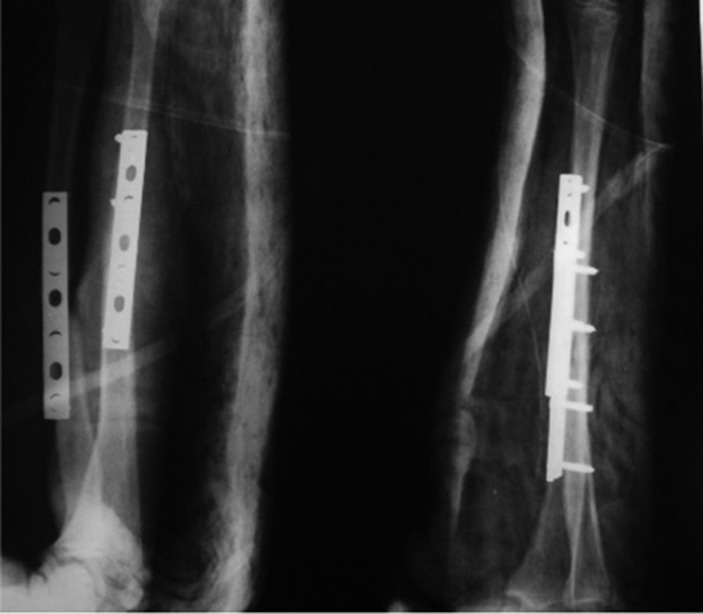
Selective fixation with 4 bicortical screws and DCP.

Infection was seen in two fractures in the conventional screw group. One superficial infection in a 56 year-old female after ulnar fixation who had diabetes and the other case was a 43 year-old male with a radius fracture without a known risk factor; whereas no infection in selected screw group occurred. The infection subsided after debridement and irrigation with antibiotics with no need to remove the plate. No change in alignment or failure of device was seen in any patient (Figure 1-4).

## 5. Discussion

Displaced diaphyseal fractures of the forearm occur from high-energy trauma, and may result in severe loss of function unless adequately treated. ORIF with DCP has been accepted as the best method of treatment for these fractures (17). There is no consensus with respect to the plate length or number of screw placement; it depends on the size of the bone fragments and degree of comminution. DCP with 6 cortices fixation is the most widely used. Regardless of the number of cortices fixated, the union rate for these fractures has been 95%-98%.

We can assume that using standard length DCP with selective 4 cortices fixation for simple diaphyseal forearm fractures which are not weight-bearing bones will be associated with the advantages of less operation time, less infection rate, and more cost-effective. In the present study the overall rate of bone union was 93.8% similar to those reported in the literature. Lindvall reported results of 75 forearm diaphyseal fractures in 53 patients and observed 97% union rate with 4 cortices screw fixation plate (13). We did not find a significant difference in the rate and time to bone union in our patients with these two methods of fixation. Perhaps, the most significant effect of 4 cortices fixation is the lower risk of refracture after plate removal. Torniquist et al. found that in plating, the torque resistance is related to the number of screws (18). Stern et al. noted that 4 cortices fixation lacked stability in oblique fractures (19). The concept of plate fixation is based on stress-shielding effect. With time, bone atrophy in the segment of bone to which a plate is applied will occur. We did not find this complication because of short term follow-up.

One interesting point of note is that the results in both groups of our patients were similar. We found that stability with 4 cortices versus 6 cortices or more was comparable to allow early gentle movement of the hand and elbow. Woo showed that with more flexible the implant is the more possible it is to decrease stress shielding and witness more callus formation (20, 21). Another important point of is that the method of selective screw fixation can be used with standard length plates. Thus, according to the type or pattern of the fracture, we can use DCP of any necessary length but with a fewer screws. Despite the similar results in both groups in our study we believe that in decision making the final judgment of the surgeon during operation is important, and when fracture stability is in question, it is advisable to fix all cortices with screws or use a longer plate to gain a stable and rigid fixation (22-24).

We think that selective screw fixation of cortices can have the following advantages: Minimal damage to cortex, decrease the time of the surgical procedure and lower refracture risk after plate removal. The main disadvantage of this method may be a less rigid fixation. We did not observe any change in alignment or position of fracture after plating in our patients. Infection occurred in two cases; both of them were in the conventional method group. With antibiotics therapy and along with debridement, both were treated; however, in one of them, nonunion of the ulnar fracture occurred. In the literature, the rate of infection after forearm fractures has been reported to be to 0 to 3.1% (3, 4, 19). The overall infection in our study was 1.6% seen after conventional plating. A late complication after plate removal from both forearm bone fractures may be refracture, which has been reported to be 4% to 25% (5, 6) This may occur at the screw tract. Although the more the number of cortices fixated via screws help to gain more stability, however, we did not find any difference in either groups. We believe that this method should be used for AO type A or B forearm diaphyseal fractures with a standard length plate. A limitation of the present study was the short follow-up time. We focused on the most important variables, such as, maintenance of stability, union time, union rate, and infection rate. Open reduction and internal fixation of diaphyseal fractures of the radius, ulna or both bones of the forearm with a standard length DCP and selective 4 cortices or 6 screw fixation had similar results. Thus, as less damage to host bone is caused we recommend DCP with 4 cortices screw fixation when the fracture pattern is simple transverse or oblique without gross contamination. To assess the rate of refracture, long-term follow-up after plate removal is needed.
